# Comprehensive Comparison Between Adjuvant Targeted Therapy and Chemotherapy for EGFR-Mutant NSCLC Patients: A Cost-Effectiveness Analysis

**DOI:** 10.3389/fonc.2021.619376

**Published:** 2021-03-25

**Authors:** Wenqian Li, Hanfei Guo, Lingyu Li, Jiuwei Cui

**Affiliations:** Cancer Center, The First Hospital of Jilin University, Changchun, China

**Keywords:** non-small-cell lung cancer, epidermal growth factor receptor-tyrosine kinase inhibitor, cost-effectiveness analysis, adjuvant therapy, chemotherapy

## Abstract

**Background:**

Chemotherapy has been the current standard adjuvant treatment for early-stage non-small-cell lung cancer (NSCLC) patients, while recent studies showed benefits of epidermal growth factor receptor-tyrosine kinase inhibitor (EGFR-TKI). We conducted a cost-effectiveness analysis to comprehensively evaluate the benefit of EGFR-TKI compared with chemotherapy for early-stage EGFR-mutant NSCLC patients after resection from the perspective of the Chinese health care system.

**Method:**

A Markov model was established. Clinical data were based on the phase 3, ADJUVANT trial, where stage II-IIIA, EGFR-mutant NSCLC patients were randomized into gefitinib group or chemotherapy group after resection. Cost parameters mainly included costs of drugs, examinations, and adverse events (AEs). Effect parameters were evaluated by quality-adjusted life year (QALY). Outcomes contained incremental cost-effective ratio (ICER), average cost-effective ratio (ACER), and net benefit. The willingness-to-pay threshold was set as 3 times per capita gross domestic product ($30,828/QALY). Sensitivity analyses were also conducted to verify the stability of the model.

**Results:**

Patients who received gefitinib had both a higher cost ($12,057.98 vs. $11,883.73) and a higher QALY (1.55 vs. 1.42) than patients who received chemotherapy. With an ICER of $1,345.62/QALY, adjuvant gefitinib was of economic benefit compared with chemotherapy. The ACER and net benefit were also consistent (gefitinib vs. chemotherapy, ACER: $7,802.30/QALY vs. $8,392.77/QALY; net benefit: $35,584.85 vs. $31,767.17). Sensitivity analyses indicated the stability of the model and the impact of utility.

**Conclusion:**

Adjuvant EGFR-TKI application for early-stage EGFR-mutant NSCLC patients was cost-effective compared with chemotherapy, which might provide a reference for clinical decision-making and medical insurance policy formulation in China.

## Background

Non-small-cell lung cancer (NSCLC) is the major type of lung cancer, among which, approximately 25% are diagnosed with early-stage NSCLC and are supposed to undergo surgical resection (1, 2). However, the high postoperative recurrence rate has a negative impact on prognosis, with approximately 30% for stage I patients and up to 75% for stage III patients (3–5). Common relapse after surgery for NSCLC patients highlights the importance of optimizing adjuvant treatment regions to eliminate residual tumors (6). Previous studies have shown that postoperative cisplatin-based chemotherapy could bring survival benefits to NSCLC patients with a 5–10% improvement in 5-year overall survival (OS) rate, furthermore, the combination of vinorelbine and cisplatin is currently the standard adjuvant treatment regimen for resected stage II–III NSCLC patients (7–10). However, the toxicity of chemotherapy reduces the compliance of patients and therapeutic efficacy (4).

Epidermal growth factor receptor (EGFR) mutation is the most common type of genomic alteration in NSCLC, with an incidence range of 10–20% in Caucasians to 50% in Asian populations (11). The efficacy of EGFR-tyrosine kinase inhibitor (EGFR-TKI) for advanced EGFR-mutant NSCLC patients is well accepted, and current studies are exploring the application of EGFR-TKI in the adjuvant setting (12). A meta-analysis confirmed the disease-free survival (DFS) benefit of adjuvant EGFR-TKI compared with both placebo (hazard ratio [HR]: 0.59, 95% confidence interval [CI]: 0.40–0.88, P = 0.009) and chemotherapy (HR: 0.42, 95%CI: 0.19–0.93, P = 0.03) for EGFR-mutant patients, however, OS analysis only showed superior tendency without significant benefit, besides patients administrated with EGFR-TKI had fewer adverse events (AEs) than patients receiving chemotherapy (risk ratio [RR]: 0.26, 95%CI: 0.18–0.38, P <0.00001) (13).

In clinical practice, despite survival benefits, cost and quality of life are also important considerations for treatment decisions. Patient’s quality of life reflects both the physical and psychological status of patients, besides, the impact of AEs is also included. Multi-dimensional assessments are not only conducive to a comprehensive evaluation and decision making but could also improve the compliance of patients. Thus, a cost-effectiveness analysis was conducted to evaluate the benefit of EGFR-TKI compared with chemotherapy as adjuvant therapy for EGFR-mutant NSCLC patients after resection, in order to select the optimal adjuvant therapy comprehensively and provide guidance for both clinical decision making and health insurance policy formulation.

## Method

### Clinical Data

The clinical data was based on a phase 3, randomized, open-label ADJUVANT trial (CTONG1104, NCT01405079), patients who underwent complete resection (R0) and diagnosed with stage II–IIIA (N1–N2), EGFR mutation-positive (exon 19 deletion or exon 21 Leu858Arg) NSCLC were eligible from multi-centers in China (14, 15). After complete resection and randomization, patients were allotted into targeted therapy group (receiving gefitinib 250 mg once daily for 24 months, oral administration) or chemotherapy group (receiving vinorelbine 25 mg/m² on days 1 and 8 plus cisplatin 75 mg/m² on day 1, every 3 weeks for four cycles, intravenous administration). After a median follow-up of 80 months, results showed patients receiving gefitinib achieved a superior DFS (median DFS: 30.8 months vs. 19.8 months, HR: 0.56, 95%CI: 0.40–0.79, P = 0.001) than those administrated with chemotherapy, while OS analysis did not show a significant difference between gefitinib and chemotherapy group (median OS: 75.5 months vs. 62.8 months, HR: 0.92, 95%CI: 0.62–1.36, P = 0.674). Besides, in terms of AEs, patients receiving gefitinib suffered from fewer AEs than patients in the chemotherapy group (AEs: 58% vs. 80%, grades 3–4: 12% vs. 48%). Detailed information was listed in **Table 1**.

**Table 1:** Clinical data.

	Gefitinib	Chemotherapy
Administration	Gefitinib (250 mg once daily) for 24 months	Vinorelbine (25 mg/m² on days 1 and 8) plus cisplatin (75 mg/m² on day 1) every 3 weeks for four cycles
Median DFS (95%CI)	30.8 (26.7–36.6)	19.8 (15.4–23.0)
HR (95%CI)	0.56 (0.40–0.79)	
P	0.001	
Median OS (95%CI)	75.5 (46.6–NC)	62.8 (45.8–NC)
HR (95%CI)	0.92 (0.62–1.36)	
P	0.674	
AE	58%	80%
Grades 3–4 AE	12%	48%

DFS, disease-free survival; OS, overall survival; HR, hazard ratio; CI, confidence interval; AE, adverse event.

### Cost-Effectiveness Parameters

**Figure 1:**
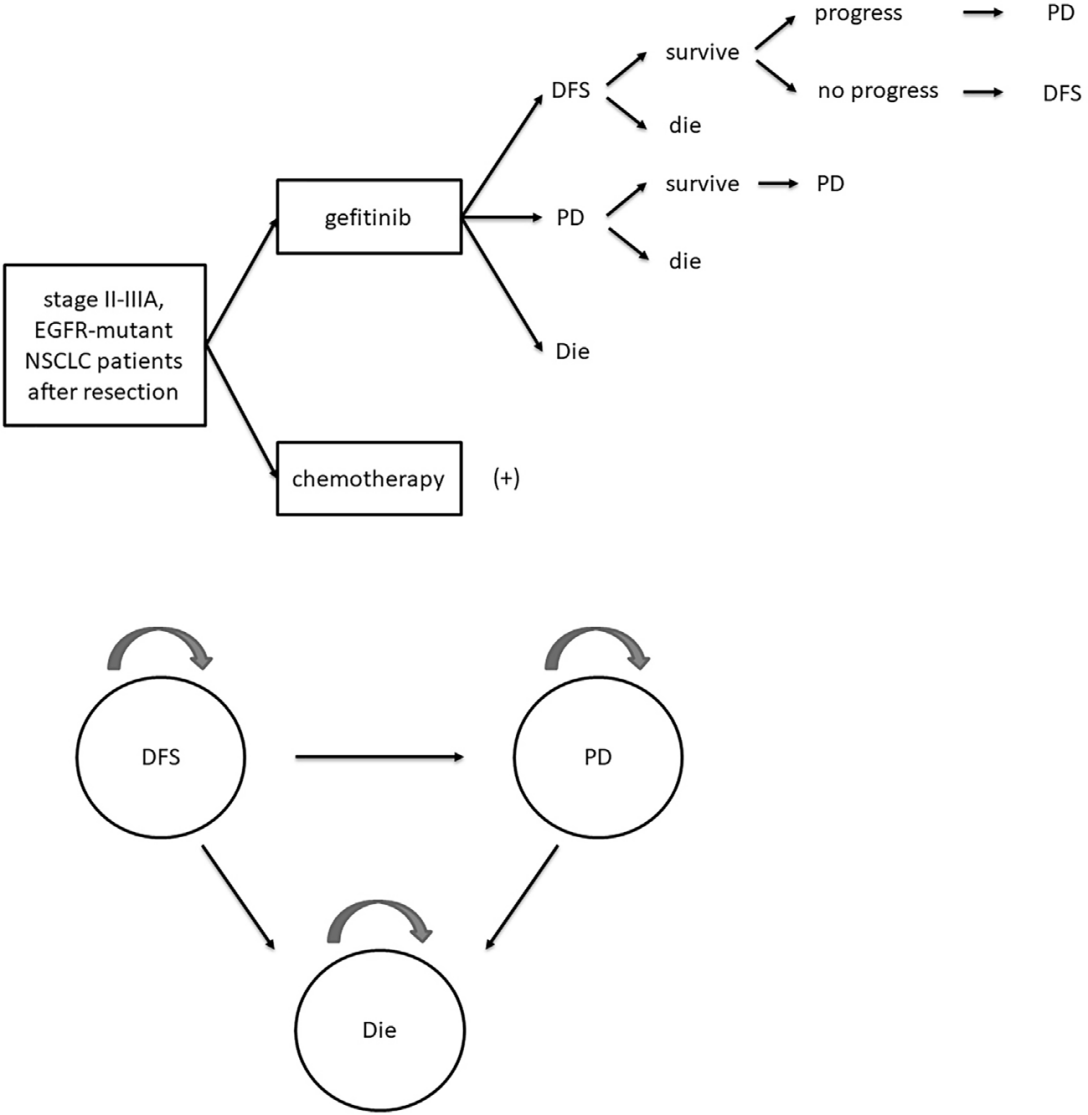
Schematic diagram of Markov model. DFS, disease-free survival; PD, progressive disease; EGFR, epidermal growth factor receptor; NSCLC, non-small-cell lung cancer.

A Markov model was established using Treeage Pro with a 21-day cycle length, a 10-year horizon, a 3% annual discount rate, and three mutually independent Markov states: DFS, progressive disease (PD), and die. All patients were in DFS state initially and transferred into other states according to progressive and survival probabilities. The structure of the Markov model was presented in **Figure 1**. Progression and survival probabilities were extracted and calculated from DFS and OS Kaplan–Meier curves respectively in the ADJUVANT trial by GetData Graph Digitizer and R software (14, 15). Fitting to the Weibull model, the following formulas were used to calculate progressive or survival probability P and transition probability at time t: P =1 − Exp(−r × t); Pt = 1 – Exp [*λ*(t − u)*_γ_* – *λ*t*_γ_*], where r represented for the progressive or survival rate, u was the cycle length, *λ* and *γ* were the scale and shape parameter separately (16).

Costs were extracted from local hospitals and published literature. For specific calculation of drug doses and costs, we assumed a typical patient with a 1.64 m height, a 65 kg weight, and a body surface area (BSA) of 1.72 m^2^ according to previous cost-effectiveness analysis evaluating chemotherapy (17). Direct medical costs of drugs (gefitinib, vinorelbine, and cisplatin), imaging examinations, laboratory tests, follow-up, supportive care, grade ≥3 AEs, and PD state were calculated as US dollars (exchange rate: 6.8409) (17, 18). Common expenses for both groups such as costs of surgery and EGFR tests were not calculated since they did not affect the cost-effectiveness results.

Effects of treatments were representative by quality-adjusted life year (QALY), which is a comprehensive evaluation index of patients’ survival period and quality of life. Health state utility parameters were extracted from published literature and were ranged from 0 to 1 with 1 representing the best physical and psychological conditions. Extracted utilities contained utilities for DFS state, including oral therapy (0.8) and intravenous therapy (0.76) respectively; PD state (0.7); death (0); and AEs of grades 3–4 (−0.0731), thereinto considering the unavailability of accurate utilities of various AEs, the average value was obtained for substitution based on published studies (19, 20). Specific utilities were adjusted based on clinical reports in the ADJUVANT trial. Cost and utility parameters were listed in **Table 2**.

**Table 2:** Baseline parameters.

Baseline parameters	Value	Specification
Cost		
Gefitinib	23.33	0.25 g ∗ 1
Vinorelbine	8.16	1 ml/10 mg ∗ 1
Cisplatin	2.80	6 ml/30 mg ∗ 1
CT Scan-Lung	54.84	Once
CT Scan-Abdomen	52.34	Once
MRI Scan-brain	91.38	Once
Electrocardiograph	3.80	Once
Echocardiography	48.60	Once
Enhanced CT Scan-Lung	134.44	Once
Enhanced CT Scan-Abdomen	248.29	Once
Bone scan	183.41	Once
PET-CT	1,154.98	Once
Abdominal ultrasonography	26.38	Once
Routine blood test	3.22	Once
Blood biochemical examination	25.29	Once
Routine urine test	4.39	Once
Coagulation test	9.36	Once
Artery blood gas	21.93	Once
Follow-up	55.60	Per cycle
Supportive care	337.50	Per cycle
AE	507.40	Per cycle
PD	1,877.25	First cycle
Utility		
DFS, oral therapy	0.80	
DFS, intravenous therapy	0.76	
PD	0.70	
Death	0.00	
AE	−0.07	

CT, computed tomography; MRI, magnetic resonance imaging; PET, positron emission tomography; AE, adverse event; PD, progressive disease; DFS, disease-free survival.

### Cost-Effectiveness and Sensitivity Analyses

The primary outcome of the study was the incremental cost-effective ratio (ICER), which is the ratio of incremental cost and incremental effect between the two groups. Secondary outcomes were the average cost-effective ratio (ACER, the ratio of average cost and average effect) and net benefit (willingness-to-pay [WTP] × effect − cost). The cost-effectiveness analysis was conducted in the perspective of the Chinese health care system and the WTP threshold was set as three times per capita gross domestic product (GDP, $30,828/QALY).

Both one-way sensitivity analysis and probabilistic sensitivity analysis were conducted by Treeage Pro. One-way sensitivity analysis was displayed as a Tornado diagram to explore the most influential factor on the Markov model. Cost parameters were evaluated with a range of 30% based on the baseline value, while a 20% range was set for both utilities and survival probabilities. Detailed information was shown in **Supplemental Table 1**. Probabilistic sensitivity analysis was conducted through Monte Carlo Simulation with 1,000 iterations, cost parameters were hypothesized to fit gamma distribution, while utilities and survival probabilities were assumed to be beta distributed (21). Results were displayed as cost-effectiveness acceptability curve and net monetary benefit acceptability curve in order to represent the cost-effective iterations with various WTP thresholds.

### Statement

Clinical data in the current manuscript were extracted from a published clinical trial (ADJUVANT trial/CTONG1104/NCT01405079) and therefore ethics approval or specific consent procedures were not required for this study.

## Results

**Figure 2:**
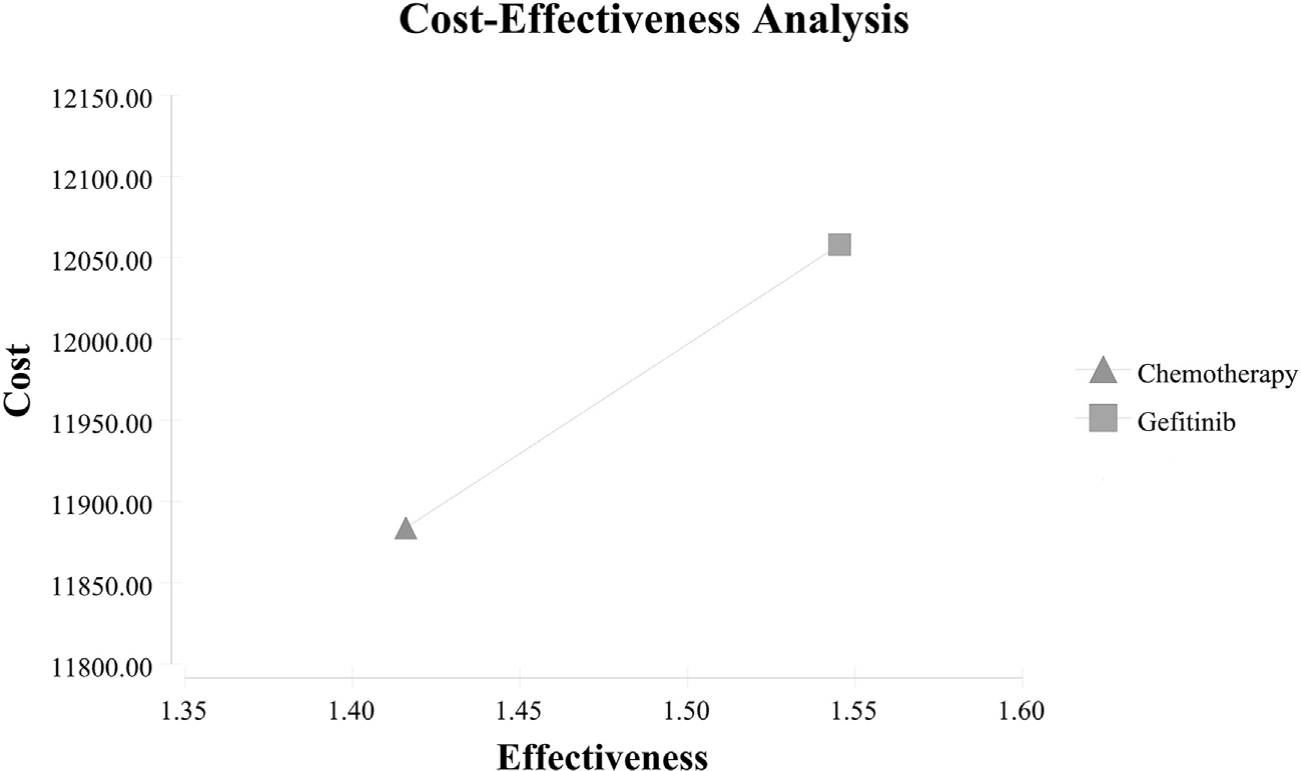
Results of cost-effectiveness analysis.

Patients receiving gefitinib achieved a better QALY than patients receiving chemotherapy (1.55 vs. 1.42) with an incremental QALY of 0.13, however, the gefitinib group also had a higher cost than the chemotherapy group ($12,057.98 vs. $11,883.73) with an incremental cost of $174.24. The ICER was $1,345.62/QALY, which indicated the administration of gefitinib was cost-effective compared to chemotherapy in the perspective of the Chinese health care system. The cost-effectiveness analysis curve was shown in **Figure 2**. As for secondary outcomes, the gefitinib group showed a lower ACER and a higher net benefit than the chemotherapy group (ACER: $7,802.30/QALY vs. $8,392.77/QALY; net benefit: $35,584.85 vs. $31,767.17). Specific results were listed in **Table 3**.

**Table 3:** Results.

	QALY	IE	Cost	IC	ICER	ACER	Net benefit
Gefitinib	1.55		12,057.98			7,802.30	35,584.85
Chemotherapy	1.42	0.13	11,883.73	174.24	1,345.62	8,392.77	31,767.17

QALY, quality-adjusted life year; IE, incremental effect; IC, incremental cost; ICER, incremental cost-effective ratio; ACER, average cost-effective ratio.

**Figure 3:**
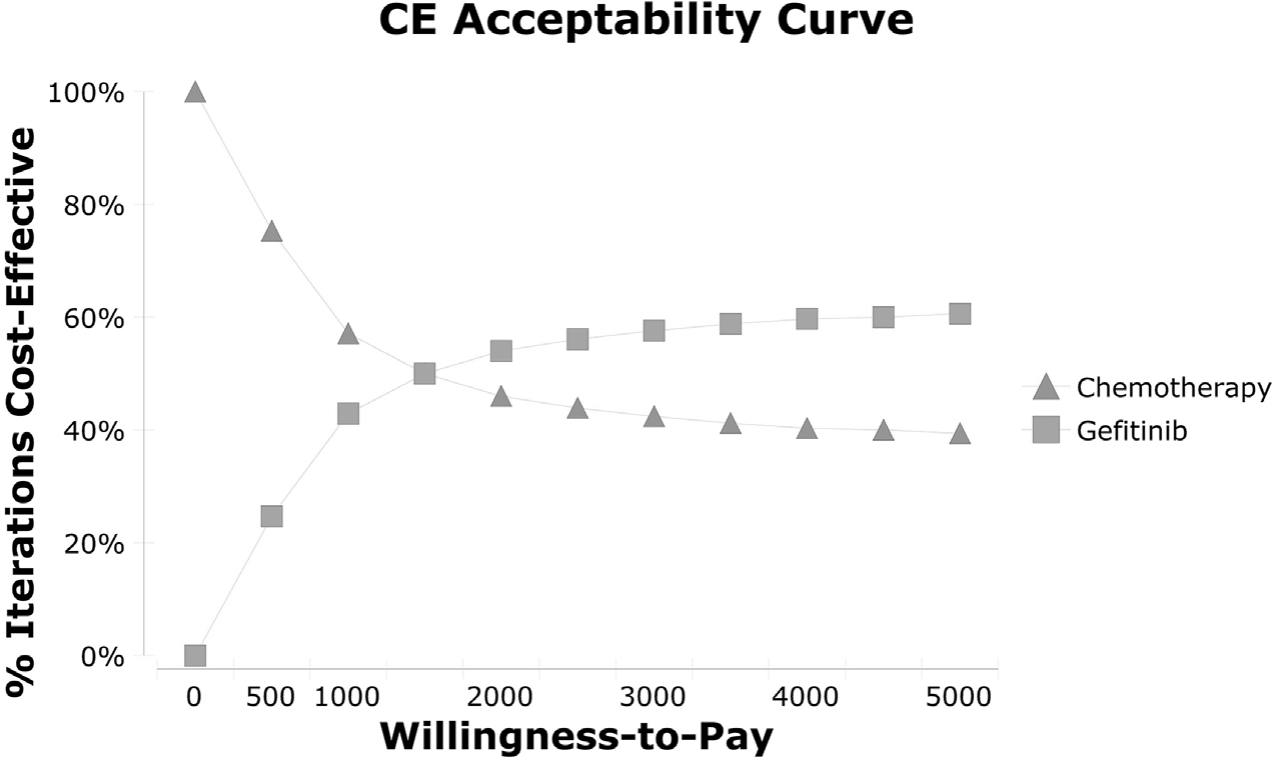
Cost-effectiveness acceptability curve. CE, cost-effectiveness.

As for sensitivity analyses, one-way sensitivity analysis showed that the utility of patients receiving gefitinib in DFS state was the most dominant influence index, followed by the utility of DFS patients receiving chemotherapy and utility of PD patients receiving gefitinib. The tornado diagram was shown in **Supplemental Figure 1**. While in terms of probabilistic sensitivity analysis, the cost-effectiveness acceptability curve showed even at a WTP of $1,500, the gefitinib group was of more economic benefit than the chemotherapy group, which was displayed in **Figure 3**. Net monetary benefit acceptability curve showed advantages gradually expanded with the increase of WTP (**Supplemental Figure 2**). Probability distributions were listed in **Supplemental Table 2**.

## Discussion

Although several clinical trials confirmed the superior DFS benefit of adjuvant EGFR-TKI over both chemotherapy and placebo, none of them showed a long-term survival benefit, in addition, it was also suggested that two years of treatment course was not conducive to the adherence of patients due to chronic AEs, hence the adjuvant application of EGFR-TKI was still controversial (22). Thus, we conducted a comprehensive evaluation of adjuvant EGFR-TKI benefits from multi-dimensions including clinical survival benefit, quality of life, and costs. According to the outcomes, administration of EGFR-TKI brought a higher cost of $174.24 and a higher QALY of 0.13, the ICER was $1,345.62/QALY, which showed prominent advantages.

Previous cost-effectiveness analyses demonstrated that the application of adjuvant chemotherapy had superior benefits compared with the observation group for early-stage NSCLC patients in the Canadian health care system perspective (23). While other cost-effectiveness studies showed economic benefits of prognostic tests in guiding adjuvant chemotherapy, which was also from the perspective of the United States and Canada health care systems (24–26). However, there was still a lack of economic evaluations on EGFR-TKI in the adjuvant setting, making this study the first cost-effectiveness analysis to comprehensively evaluate the benefit of adjuvant EGFR-TKI therapy for early-stage EGFR-mutant NSCLC patients.

Currently, only advanced EGFR mutation-positive NSCLC patients receiving first-generation EGFR-TKI are included in the Chinese medical insurance policy. Considering the consistent DFS, safety, and cost-effectiveness benefits of first-generation EGFR-TKI application for early-stage EGFR mutation-positive NSCLC patients, it is suggested that the reimbursement policy could be further expanded. In addition, the cost of gefitinib was based on the ADJUVANT trial, while the cost of domestic gefitinib was cheaper, which could further expand the benefits.

In spite of the positive outcomes, further explorations and developments are still required in this field. Firstly, since the survival benefit of the third-generation EGFR-TKI, osimertinib for advanced EGFR-mutant NSCLC patients has been verified, studies are also exploring the efficacy of osimertinib in the adjuvant setting for EGFR-mutant NSCLC patients after complete resection (27). The phase 3 ADAURA (NCT02511106) trial demonstrated that patients receiving osimertinib achieved a significant superior DFS compared with those receiving placebo (stage II–IIIA patients: HR, 0.17; 99.06%CI, 0.11–0.26; P <0.001; stage IB–IIIA patients: HR, 0.20; 99.12%CI, 0.14–0.30; P <0.001) (28). We did not include osimertinib in the cost-effectiveness analysis due to the immature survival data, further exploration could be conducted with mature data. Secondly, although EGFR-TKI monotherapy could reduce AEs, considering tumor heterogeneity and the efficacy of other treatment regimens, including chemotherapy and radiotherapy, different combination therapies should also be further investigated for assessing the optimal adjuvant therapy (22). Thirdly, there is still a lack of relevant studies for treatments after resistance to EGFR-TKI adjuvant therapy, which should be further explored as well (5).

The main limitation of the study was that the outcomes were restricted to geographical regions and populations. Despite the phase 2, single-arm, SELECT trial also showed efficacy of adjuvant EGFR-TKI therapy based on major non-Asian population (5-year DFS rate: 56%, 5-year OS rate: 86%), studies showed that both EGFR mutation rate and therapeutic efficacy of EGFR-TKI are related to ethnicity with distinctive clinicopathologic characteristics (3, 29). Both the clinical data and cost parameters of this cost-effectiveness analysis were based on Chinese populations, thus it is not suitable to generalize the outcomes to Caucasians or other populations.

## Conclusion

In the cost-effectiveness analysis, we comprehensively evaluated the benefit of adjuvant EGFR-TKI application for early-stage EGFR mutation-positive NSCLC patients by synthesizing clinical survival data, quality of life and cost parameters. The ICER was $1,345.62/QALY and demonstrated economic benefits from the perspective of the Chinese health care system. Our results could further propel the development of precision treatment, and provide a reference for clinical decision-making and medical insurance policy formulation in China.

## Data Availability Statement

The original contributions presented in the study are included in the article/**Supplementary Material**. Further inquiries can be directed to the corresponding author.

## Ethics Statement

Clinical data in the current manuscript were extracted from published clinical trial (ADJUVANT trial/CTONG1104/NCT01405079) and therefore ethics approval or specific consent procedures were not required for this study.

## Author Contributions

Guarantor of integrity of the entire study: JC. Study concepts and design: JC and WL. Literature research: WL and HG. Data analysis: WL and LL. Manuscript preparation: WL and JC. Manuscript editing: JC. All authors contributed to the article and approved the submitted version.

## Funding

This work was supported by Nation Key Research and Development Program of China (Grant No. 2016YFC1303800).

## Conflict of Interest

The authors declare that the research was conducted in the absence of any commercial or financial relationships that could be construed as a potential conflict of interest.
