# Supplementary material for: Comprehensive Comparison Between Adjuvant Targeted Therapy and Chemotherapy for EGFR-Mutant NSCLC Patients: A Cost-Effectiveness Analysis
Source: Front Oncol. 2021 Mar 25;11:619376. doi: 10.3389/fonc.2021.619376 (PMC8027108; doi:10.3389/fonc.2021.619376)
Supplement: Supplementary file 1 [file DataSheet_1.docx]

**Supplemental Fig 1.** Tornado diagram. GUDFS: utility of disease-free survival state in gefitinib group; CUDFS: utility of disease-free survival state in chemotherapy group; GUPD: utility of progressive disease state in gefitinib group; CUPD: utility of progressive disease state in chemotherapy group; GCDFS: cost of disease-free survival state in gefitinib group; CCDFS: cost of disease-free survival state in chemotherapy group; GCPD: cost of progressive disease state in gefitinib group; CCPD: cost of progressive disease state in chemotherapy group; GSDFS: progressive probability of gefitinib group; CSDFS: progressive probability of chemotherapy group; GSPD: survival probability of gefitinib group; CSPD: survival probability of chemotherapy group.

**Supplemental Fig 2.** Net monetary benefit acceptability curve. NMB: net monetary benefit.

**Supplemental table 1. Sensitivity analysis**

|  | baseline value | lower limit | upper limit |
| --- | --- | --- | --- |
| **cost (first cycle)** |  |  |  |
| DFS state (gefitinib group) | 2102.27 | 1471.59 | 2732.94 |
| DFS state (chemotherapy group) | 2674.51 | 1872.16 | 3476.86 |
| PD state | 1877.25 | 1314.08 | 2440.43 |
| **utility (per cycle)** |  |  |  |
| DFS state (gefitinib group) | 0.0495 | 0.0396 | 0.0593 |
| DFS state (chemotherapy group) | 0.0453 | 0.0362 | 0.0544 |
| PD state | 0.0438 | 0.0350 | 0.0525 |
| **probability (first cycle)** |  |  |  |
| progressive (gefitinib group) | 0.0124 | 0.0099 | 0.0149 |
| progressive (chemotherapy group) | 0.0074 | 0.0059 | 0.0088 |
| survival (gefitinib group) | 0.0062 | 0.0049 | 0.0074 |
| survival (chemotherapy group) | 0.0058 | 0.0047 | 0.0070 |

DFS: disease-free survival; PD: progressive disease.

**Supplemental table 2. Results of probabilistic sensitivity analysis**

| Parameters | gefitinib | | chemotherapy | |
| --- | --- | --- | --- | --- |
|  | mean | std deviation | mean | std deviation |
| cost | 12057.9700 | 0.1839 | 11883.7358 | 0.0760 |
| effect | 1.5528 | 0.2447 | 1.4270 | 0.2201 |
| NMB | 35819.7605 | 7542.2382 | 32106.5044 | 6784.3170 |
| DFS cost | 1635.2254 | 391.2778 | 1610.7050 | 389.6721 |
| DFS utility | 0.0497 | 0.0083 | 0.0457 | 0.0077 |
| PD cost | 1866.5640 | 468.3541 | 1881.1448 | 470.8502 |
| PD utility | 0.0439 | 0.0071 | 0.0435 | 0.0069 |
| progressive probability | 0.0124 | 0.0021 | 0.0073 | 0.0012 |
| survival probability | 0.0062 | 0.0010 | 0.0059 | 0.0009 |
