# Supplementary figures and images for: Comprehensive Comparison Between Adjuvant Targeted Therapy and Chemotherapy for EGFR-Mutant NSCLC Patients: A Cost-Effectiveness Analysis
Source: Front Oncol. 2021 Mar 25;11:619376. doi: 10.3389/fonc.2021.619376 (PMC8027108; doi:10.3389/fonc.2021.619376)

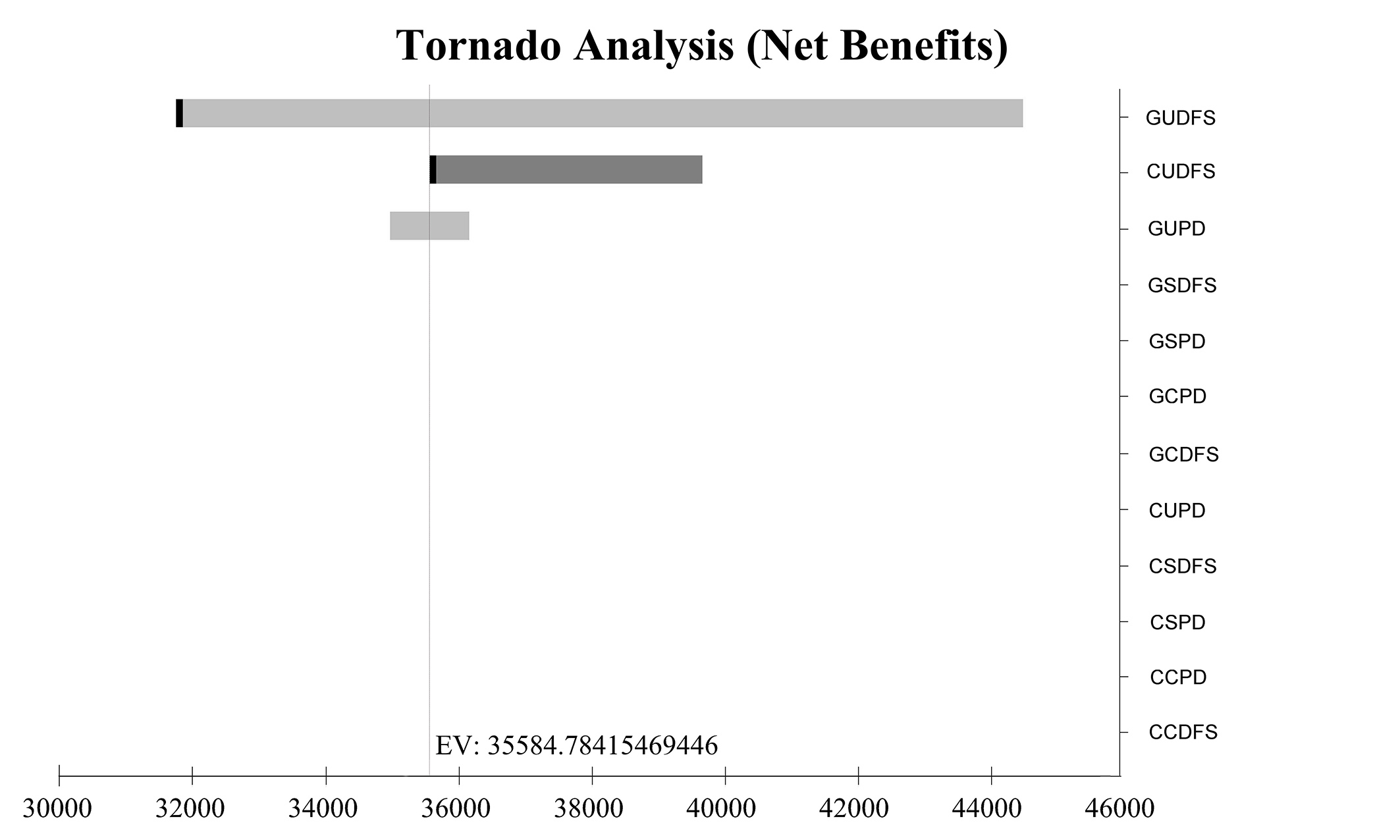

Supplement: Supplementary file 2 [file Image_1.jpeg]

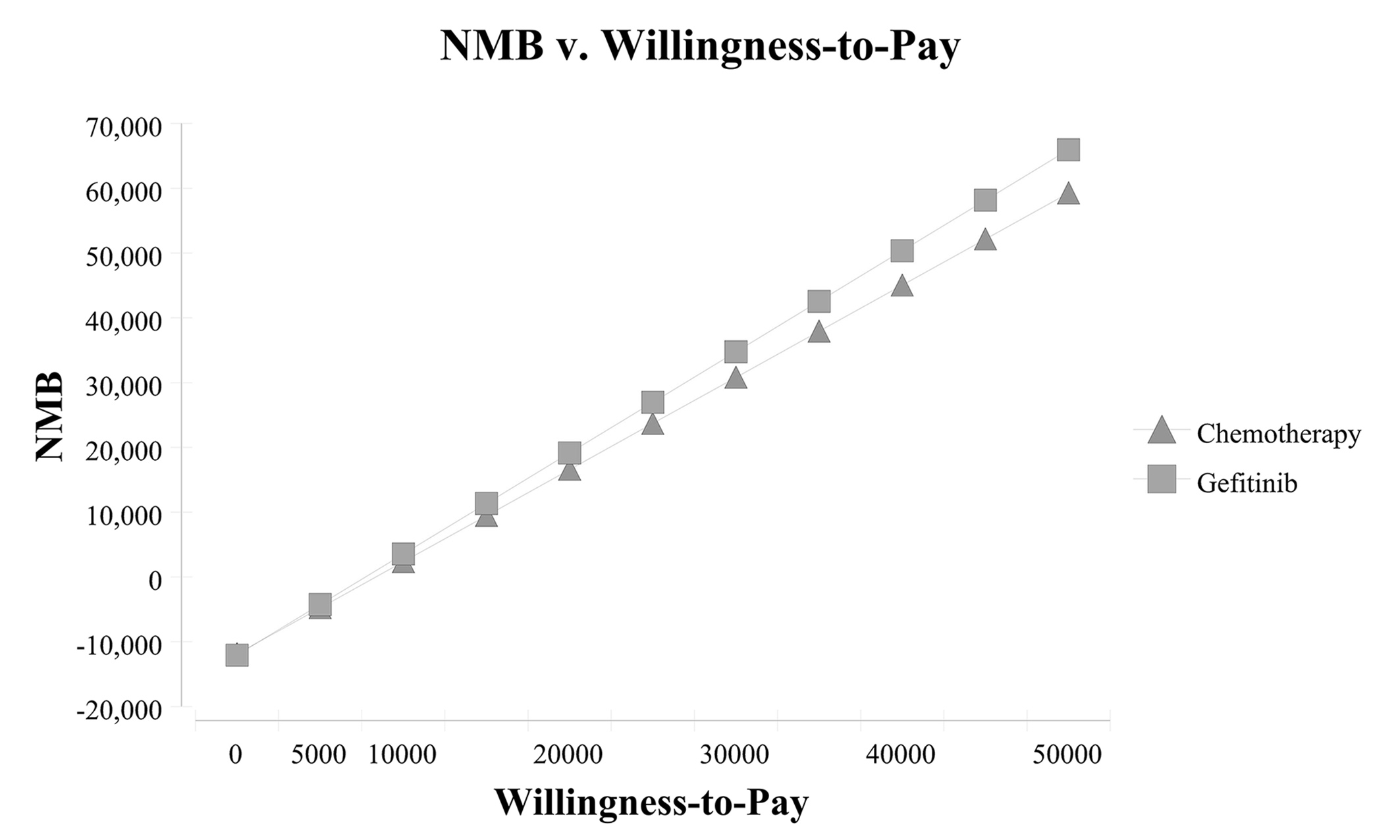

Supplement: Supplementary file 3 [file Image_2.jpeg]
